# Impact and Cost of Scaling Up Voluntary Medical Male Circumcision for HIV Prevention in the Context of the New 90-90-90 HIV Treatment Targets

**DOI:** 10.1371/journal.pone.0155734

**Published:** 2016-10-26

**Authors:** Katharine Kripke, Jason Reed, Catherine Hankins, Gregory Smiley, Catey Laube, Emmanuel Njeuhmeli

**Affiliations:** 1 Project SOAR (Supporting Operational AIDS Research), Avenir Health, Washington, District of Columbia, United States of America; 2 Jhpiego, Washington, District of Columbia, United States of America; 3 Department of Global Health and Amsterdam Institute for Global Health and Development, University of Amsterdam, Amsterdam, the Netherlands; 4 Joint United Nations Programme on HIV/AIDS Regional Support Team, Eastern and Southern Africa, Johannesburg, South Africa; 5 Office of the U.S. Global AIDS Coordinator, Washington, District of Columbia, United States of America; 6 United States Agency for International Development, Washington, District of Columbia, United States of America; World Health Organization, SWITZERLAND

## Abstract

**Background:**

The report of the Joint United Nations Programme on HIV/AIDS (UNAIDS) for World AIDS Day 2014 highlighted a Fast-Track Strategy that sets ambitious treatment and prevention targets to reduce global HIV incidence to manageable levels by 2020 and end the AIDS epidemic by 2030. The 90-90-90 treatment targets for 2020 call for 90% of people living with HIV to know their HIV status, 90% of people who know their status to receive treatment, and 90% of people on HIV treatment to be virally suppressed. This paper examines how scale-up of voluntary medical male circumcision (VMMC) services in four priority countries in sub-Saharan Africa could contribute to ending the AIDS epidemic by 2030 in the context of concerted efforts to close the treatment gap, and what the impact of VMMC scale-up would be if the 90-90-90 treatment targets were not completely met.

**Methods:**

Using the Goals module of the Spectrum suite of models, this analysis modified ART (antiretroviral treatment) scale-up coverage from base scenarios to reflect the 90-90-90 treatment targets in four countries (Lesotho, Malawi, South Africa, and Uganda). In addition, a second scenario was created to reflect viral suppression levels of 75% instead of 90%, and a third scenario was created in which the 90-90-90 treatment targets are reached in women, with men reaching more moderate coverage levels. Regarding male circumcision (MC) coverage, the analysis examined both a scenario in which VMMCs were assumed to stop after 2015, and one in which MC coverage was scaled up to 90% by 2020 and maintained at 90% thereafter.

**Results:**

Across all four countries, scaling up VMMC is projected to provide further HIV incidence reductions in addition to those achieved by reaching the 90-90-90 treatment targets. If viral suppression levels only reach 75%, scaling up VMMC leads to HIV incidence reduction to nearly the same levels as those achieved with 90-90-90 without VMMC scale-up. If only women reach the 90-90-90 targets, scaling up VMMC brings HIV incidence down to near the levels projected with 90-90-90 without VMMC scale-up. Regarding cost, scaling up VMMC increases the annual costs during the scale-up phase, but leads to lower annual costs after the MC coverage target is achieved.

**Conclusions:**

The scenarios modeled in this paper show that the highly durable and effective male circumcision intervention increases epidemic impact levels over those of treatment-only strategies, including the case if universal levels of viral suppression in men and women are not achieved by 2020. In the context of 90-90-90, prioritizing continued successful scale-up of VMMC increases the possibility that future generations will be free not only of AIDS but also of HIV.

## Introduction

The report of the Joint United Nations Programme on HIV/AIDS (UNAIDS), *UNAIDS 2016–2021 Strategy*: *On the Fast Track to End AIDS* [1], for World AIDS Day 2014 highlighted UNAIDS’ Fast-Track Strategy to end AIDS by 2030. To achieve this goal, the number of new HIV infections and AIDS-related deaths will need to decline by 90% compared to 2010 [1]. HIV treatment has dramatically extended the lifespan of people living with HIV (PLHIV), preventing deaths and onward HIV transmission, and modeling and epidemiological studies have suggested that HIV prevention programs have sharply lowered rates of new HIV infections in some countries [2]. Fast-track sets both treatment and primary prevention targets that, if achieved, will drastically reduce new infections over the next five years, reducing HIV to manageable levels by 2020 in order to make the 2030 goal attainable.

The 90-90-90 treatment targets set for 2020 call for 90% of PLHIV to know their HIV status, 90% of people who know their status to receive treatment, and 90% of people on HIV treatment to be virally suppressed. This would translate to 73% of PLHIV achieving viral suppression by 2020. Currently, an estimated 51% of adults living with HIV in sub-Saharan Africa know their HIV status, approximately 43% of adults living with HIV are receiving antiretroviral therapy, and an estimated 32% of adults living with HIV are virally suppressed [3]. Given that 84% of people who know their HIV status are on treatment and 74% of people on treatment are virally suppressed, currently the biggest gap is knowledge of HIV status. Furthermore, the Fast-Track Strategy goals are predicated upon reaching equally essential coverage targets for primary prevention, including 80% male circumcision prevalence in priority settings in sub-Saharan Africa by 2020. The UNAIDS Fast-Track report [1] further defined fast-track requirements, including an additional 27 million male circumcisions (roughly 90% of males ages 10–29) in priority countries, 3 million people on PrEP (pre-exposure prophylaxis), 30 condoms per man per year, and an array of other targets needed by 2020. The treatment and prevention gaps will have to be addressed in order to reduce AIDS-related deaths and slow the rate of new infections to reach the 2030 goals.

Randomized controlled trials have shown that voluntary medical male circumcision (VMMC) reduces males’ risk of HIV acquisition by about 60% [4], and follow-on studies have shown that this level of protection increases over time to reach 74% [5]. VMMC programs offer an important entry point to both HIV prevention and treatment for men. They include promotion of condoms and safer sexual practices, treatment for sexually transmitted infections, and optional HIV testing and counseling. Men who test HIV-positive in VMMC settings are referred for HIV care and treatment. Program evaluation data have shown a sharp increase in HIV case identification and treatment initiation in men due to the introduction of VMMC services [6], demonstrating the potential of the VMMC platform not only as a primary prevention service but also as a critical contributor to 90-90-90 treatment goals specifically for males.

Antiretroviral treatment (ART) leading to viral suppression has been shown to reduce HIV transmission by 96% [7], and recent trials have shown the clinical benefits for individuals of early treatment [8,9]. In September 2015 the World Health Organization released guidelines recommending initiation of ART for all people testing positive for HIV, regardless of CD4 count [10]. If countries now scale up strategies to offer HIV testing with an immediate offer of ART, they will be on the road to achieving the aspirational 90-90-90 targets. However, as with prevention goals, progress in meeting treatment goals has been and will likely continue to be uneven across countries, between sexes, and among the diverse population groups at greatest risk of HIV exposure or transmission. This variability underscores the importance of preventing new infections through all available effective interventions in order to mitigate what will be an increasingly urgent challenge to treatment access.

This paper examines how VMMC scale-up in four priority countries in sub-Saharan Africa could contribute to ending the AIDS epidemic by 2030 in the context of concerted efforts to close the treatment gap. The four countries—Lesotho, Malawi, South Africa, and Uganda—were chosen to reflect diverse sizes and stages of HIV epidemics and differing levels of program coverage. In addition, the paper examines a scenario in which treatment target attainment is 90-90-75, equating to overall viral suppression of 61% by 2020, rather than 73%, in light of the possibility that current logistical, behavioral, and other challenges to adherence will not be fully overcome. The paper also looks at the impact of scaling up VMMC in a context where women are more likely than men to get tested and access ART. Historically, women are more likely to learn their HIV status and to do so earlier in the course of their disease, because they may be routinely offered HIV testing in pregnancy when they still may be asymptomatic, with the result that they can initiate and reap the benefits of treatment earlier [8,9].

## Methods

IRB clearance was not required for this study, since patient records were not collected or reviewed.

### Spectrum/Goals Models

All modeling was performed using the Goals module within the Spectrum suite of modeling tools [11]. Base Goals files were prepared for each country (Lesotho, Malawi, South Africa, and Uganda) based on publicly available data from surveys such as the Demographic and Health Surveys (DHS). Input parameters and curve fits have been provided in S1 and S2 Appendices and S1 Fig. The historical ART and PMTCT (prevention of mother-to-child transmission of HIV) service statistics were imported from the nationally validated 2015 Spectrum/AIM (AIDS Impact Model) [12] file for each country.

The Goals model does not allow the user to specify whether circumcisions are conducted among HIV-negative males only or all males; male circumcision (MC) coverage is represented in the model as being among all males regardless of HIV status and includes both medical and traditional circumcisions. “Reduction in male susceptibility when circumcised” was set to 60%, and “reduction in male infectiousness when circumcised” was set to zero.

### ART Scenarios

The modeling employed three scenarios for ART scale-up: 90-90-90, 90-90-75, and 90-90-90F (for females). Input parameters for treatment have been provided in S3 Appendix. In the 90-90-90 scenario, ART scale-up from 2015 was modified from the base file to reflect the 90-90-90 treatment targets as described elsewhere [13]. The 90-90-75 scenario was the same as the 90-90-90 scenario, except that the “ART effect” parameter (ratio of infectiousness with ART to without ART), which was used to model the level of viral suppression, was kept at base levels (ART effect 0.25—the default value—equivalent to 75% viral suppression for all on ART) for every year of the model. In the 90-90-90F scenario, the 90-90-90 ART targets were used for women, while the base targets from the nationally validated Spectrum/AIM files were used for men. In each country, the male ART coverage was scaled up more modestly than in the 90-90-90 scenario, but these targets varied by country. It is not possible to specify differences in the “ART effect” by sex in Goals, so this parameter was the same for both sexes, reflecting the assumption that men and women who are on ART have a similar quality of care and levels of adherence.

### VMMC Scenarios

The authors used VMMC program data and the DMPPT 2.1 (Decision Makers’ Program Planning Tool) model [13] to estimate the MC coverage among males ages 15–49 for each country in 2015. MC coverage was increased using linear interpolation between the base coverage levels and 2015 levels. The base coverage level was the MC prevalence among males ages 15–49 from the last DHS survey in each country before the start of their VMMC program [14–17], except that the MC prevalence for Lesotho and Malawi was divided by two to account for 50% of traditional circumcisions being incomplete or misreported [18]. For the “no VMMC” scenarios, the MC coverage level in 2015 was linearly interpolated back down to the base level by 2050, in order to represent termination of the VMMC program after 2015 and only traditional circumcisions continuing. For the VMMC scale-up scenarios (“+VMMC”), MC coverage levels were scaled up to 90% using linear interpolation between 2015 and 2020 and maintained at 90% thereafter.

### Unit Costs

VMMC unit costs were derived as described elsewhere [13]. ART unit costs were the same as those used in the Fast-Track analysis [19] (Table 1). All cost estimates used represent fully loaded costs including staff time, commodities, facility costs, equipment, laboratory costs, and program management.

**Table 1:** Unit Costs Used in the Study.

Country	VMMC Unit Cost ($US)	Annual Per Person ART Cost ($US)
Lesotho	91	384
Malawi	76	157
South Africa	152	277
Uganda	82	199
Median Sub-Saharan Africa	88	304
Average Sub-Saharan Africa	100	455

### Analysis

All model outputs were exported using the Spectrum export tool [20]. Combined annual VMMC and ART program costs were calculated from the annual numbers of people receiving VMMC and ART, exported from the models, and multiplied by the respective unit cost for each intervention for each country. For the “no VMMC” scenarios, the annual VMMC costs were zero.

## Results

### Impact and cost of scaling up VMMC in the context of achieving the 90-90-90 HIV treatment goals

**Fig 1:**
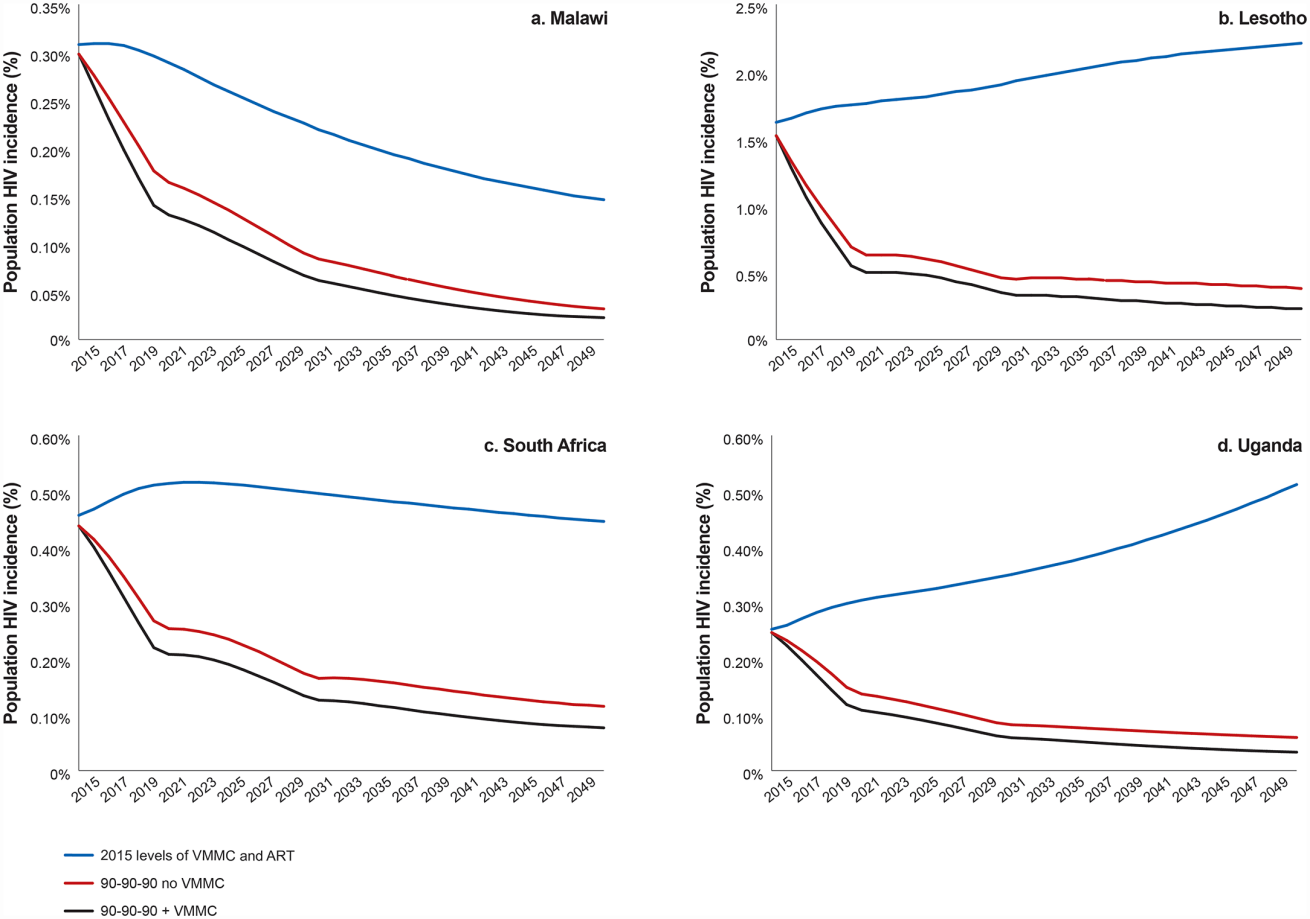
HIV incidence with and without scaling up ART and VMMC. Incidence shown is among the total population, males and females, all ages.

In addition to the reductions in HIV incidence that may be achieved by reaching the 90-90-90 treatment targets, scaling up VMMC to reach 90% MC coverage over five years and then maintaining that level of coverage is projected to provide further HIV incidence reductions (Fig 1). This is true across the four countries modeled, regardless of the starting HIV incidence level (Lesotho, 1.52%; Malawi, 0.30%; South Africa, 0.41%; Uganda, 0.25%) or estimated MC coverage (Lesotho, 39%; Malawi, 14%; South Africa, 55%; Uganda, 49%) in 2015.

**Fig 2:**
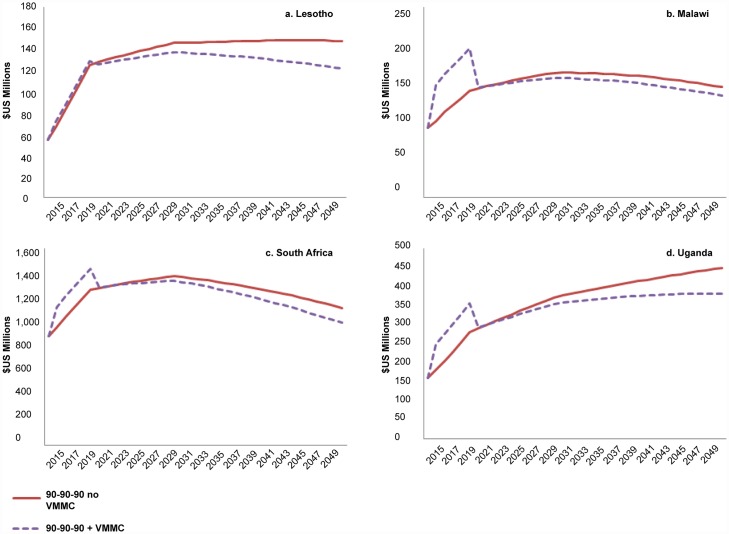
Annual cost ($US) with 90-90-90 ART scenario, with and without scaling up VMMC. Cost shown is the annual combined cost of providing VMMC and ART.

The annual cost of providing both VMMC and ART is substantially higher initially during the VMMC scale-up phase in Malawi (Fig 2b), where both MC coverage and HIV incidence in 2015 are relatively low. In Lesotho, where the estimated MC coverage in 2015 is higher, and the HIV incidence in 2015 is estimated to be 1.39% (the highest of the four countries), the cost of scaling up VMMC is incremental compared with the cost of scaling up ART alone (Fig 2a). In South Africa and Uganda, where MC coverage is high and HIV incidence is low in 2015, the addition of VMMC moderately increases the cost of the program during the VMMC scale-up phase (Fig 2c and 2d). These trends are maintained even when using the same costs for ART and VMMC across all four countries. After achieving the 90% MC coverage target while continuing circumcisions to maintain 90% coverage, the annual cost of VMMC and ART is lower than the cost of ART alone (if VMMC were not provided after 2015), because the annual numbers of people requiring treatment are lower when VMMC is scaled up (S1 Table).

### Impact and cost of scaling up VMMC if 90% viral suppression among those on ART is not possible

**Fig 3:**
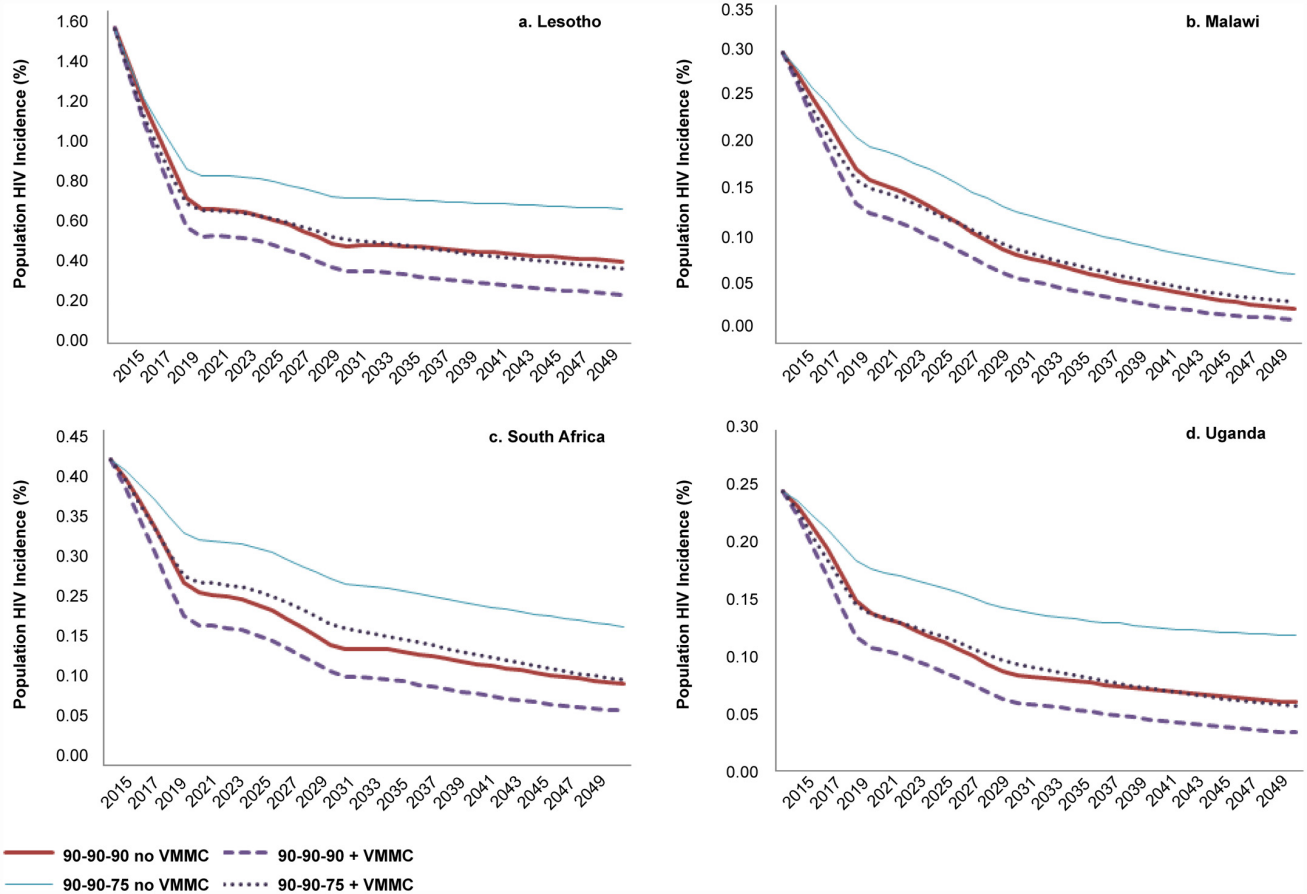
HIV incidence with 90-90-90 and 90-90-75 ART scenarios, with and without scaling up VMMC. Incidence shown is among the total population, males and females, all ages.

Even if the aspirational diagnosis and treatment initiation targets are achieved (the first two “90s”), it may be difficult to improve viral suppression. If it is not possible to increase viral suppression above 2015 levels (90-90-75 scenario), the annual HIV incidence is higher than if the 90-90-90 targets are perfectly achieved (Fig 3). Scaling up VMMC in the context of 90-90-75 leads to an HIV incidence reduction to nearly the same levels as those achieved with 90-90-90 alone.

**Fig 4:**
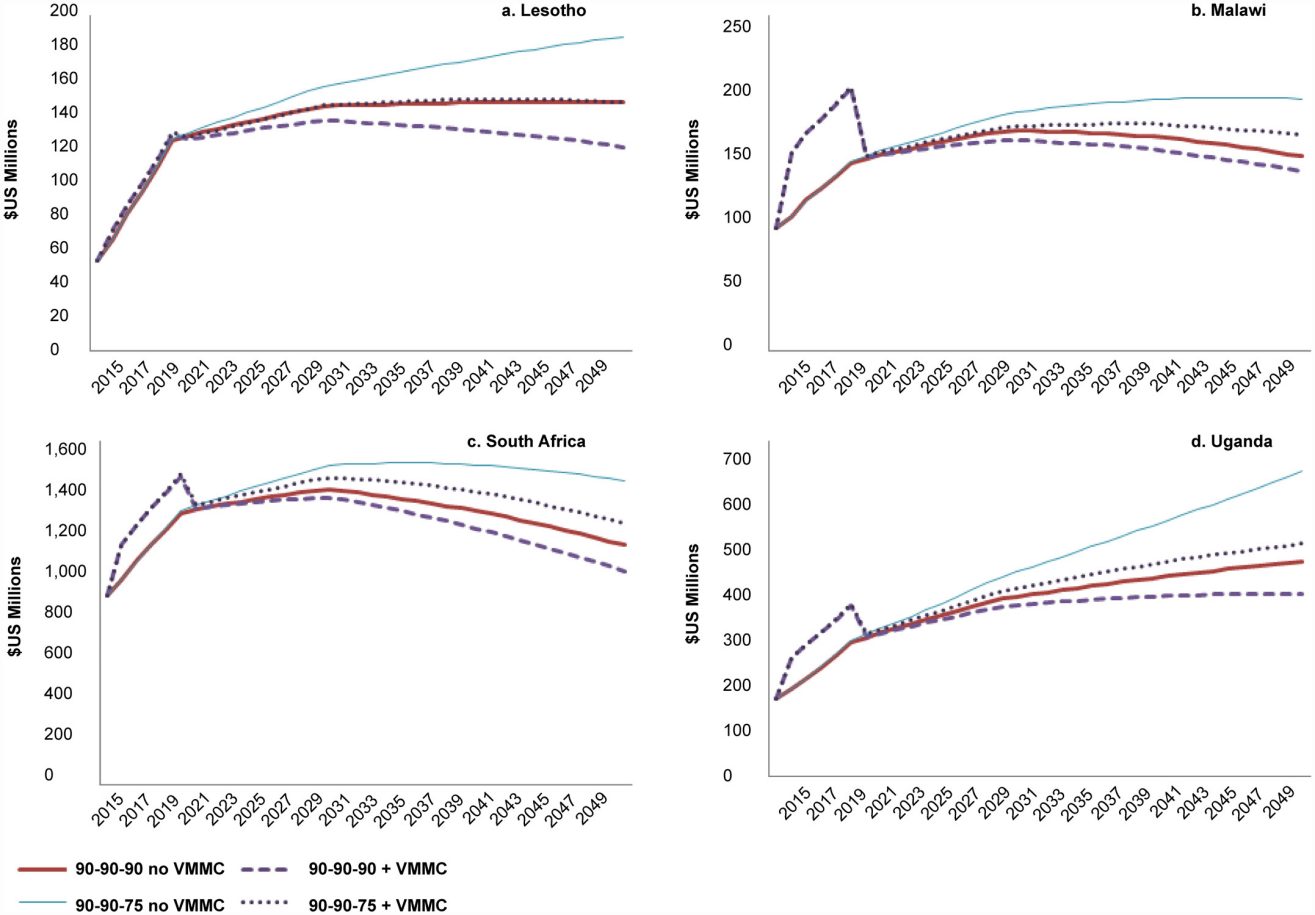
Annual cost ($US) with 90-90-90 and 90-90-75 ART scenarios, with and without scaling up VMMC. Cost shown is the annual combined cost of providing VMMC and ART.

Likewise, from a cost perspective, comparing the 90-90-75 +VMMC to the 90-90-90 no VMMC lines in Fig 4, if viral suppression remains at 75% after 2015, the annual costs after 90% MC coverage is reached are equal to or only slightly higher than the cost of 90-90-90 without scaling up VMMC. Furthermore, the inclusion of VMMC scale-up leads to a greater reduction in annual cost with the 90-90-75 scenario than with the 90-90-90 scenario.

### Impact and cost of scaling up VMMC if the 90-90-90 HIV treatment targets are reached among women but not among men

**Fig 5:**
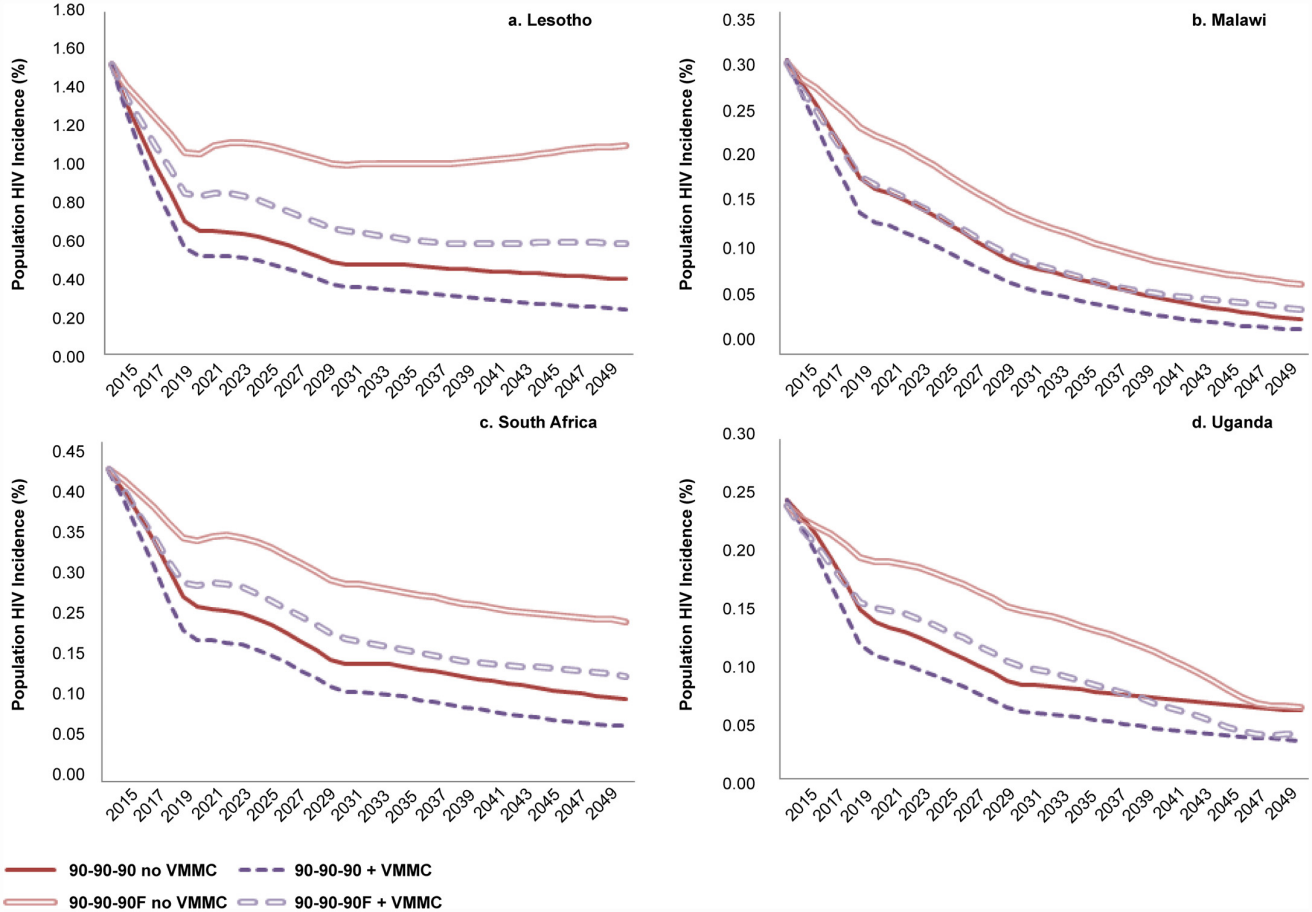
HIV incidence with 90-90-90 and 90-90-90F ART scenarios, with and without scaling up VMMC. Incidence shown is among the total population, males and females, all ages.

Because women access ART at higher rates than men in these countries, the 90-90-90 targets may be more difficult to achieve among men than among women. At the same time, VMMC services have been successful in many settings in attracting men. Therefore, we examined a scenario (“90-90-90F”), in which the 90-90-90 HIV treatment targets are met for women, but targets for men are derived from the 2015 national Spectrum/AIM files for each country, lower than the 90-90-90 targets. In the 90-90-90F scenarios, the scale-up of VMMC brings HIV incidence back down to near the levels projected in the 90-90-90 scenario without VMMC (Fig 5).

**Fig 6:**
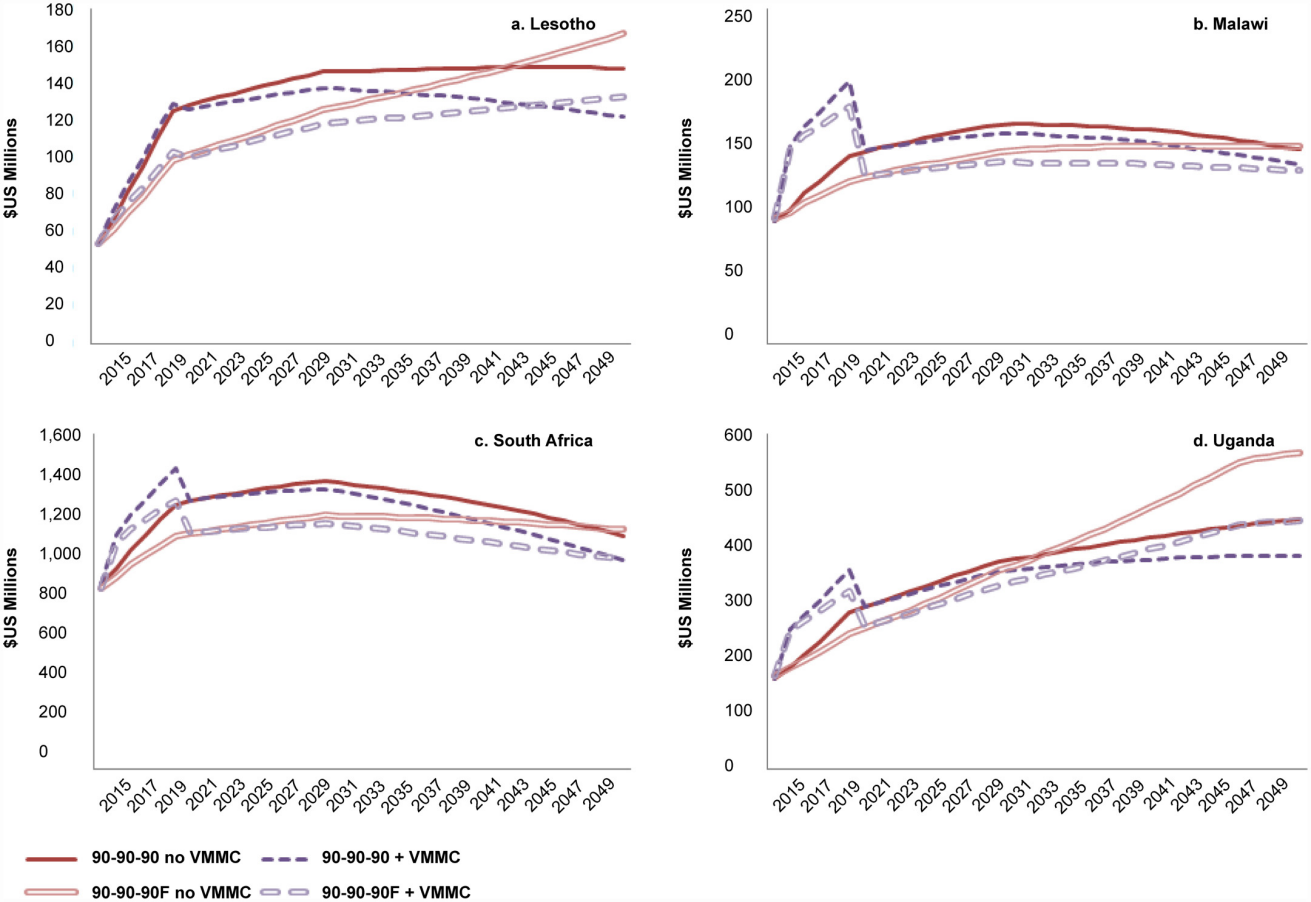
Annual cost ($US) with 90-90-90 and 90-90-90F ART scenarios, with and without scaling up VMMC. Cost shown is the annual combined cost of providing VMMC and ART.

The annual costs for the 90-90-90F scenarios tend to be lower in the early years compared with the 90-90-90 scenarios, because fewer people are on ART (Fig 6). As in the other scenarios examined, the addition of VMMC scale-up increases the annual costs during the scale-up phase but leads to lower annual costs after the MC coverage target is achieved, compared with the scenarios in which VMMC stops after 2015. In Lesotho and Uganda, the 90-90-90F costs in later years are higher than those of the corresponding 90-90-90 scenarios, and the addition of VMMC (“90-90-90F + VMMC”) brings the costs back down below the level of the 90-90-90 scenario without VMMC (“90-90-90 no VMMC”).

## Discussion

### Impact

Compared to the epidemic impact of scaling up ART to 90-90-90 levels, three scenarios that also include VMMC scale-up demonstrated additional reductions in HIV incidence and lower long-term annual program costs in models applied to Lesotho, Malawi, South Africa, and Uganda. In the first (90-90-90) scenario, combined scale-up of ART and VMMC affords greater reductions in HIV incidence than would be achieved by ART scale-up alone in all four countries. In the second (90-90-75) scenario, should viral suppression rates of those on ART remain at 2015 levels of 75%—even in the context of nearly universal early diagnosis and treatment initiation in both men and women—VMMC scale-up restores incidence reduction levels compared to the 90-90-90 scenario (without scaling up VMMC) in all four countries. In the third (90-90-90F) scenario, if 90-90-90 treatment targets are achieved only in women by 2020—but rates of diagnosis and treatment in men follow the more modest scale-up targets outlined in the national AIM files—VMMC scale-up leads to incidence levels that are nearly as low as the scenario reaching the 90-90-90 goals for both men and women, without scaling up VMMC.

Scenarios 2 and 3 (90-90-75 and 90-90-90F, respectively) are intended to reflect the impact under conditions in which viral suppression targets are not fully realized by 2020. The 90-90-75 scenario reflects full achievement of HIV diagnosis and treatment initiation targets by 2020, but holds transmission risk (as a proxy of viral suppression among those on ART) at 2015 levels in men and women, leading to an overall 61% viral suppression rate among all HIV-positive individuals. This projection was based on an assumption that behaviors related to testing and treatment initiation, as one-time events, might be more readily achieved than those required for viral suppression, which entail lifelong daily adherence once ART is started. Note that the 61% viral suppression scenario could have been modeled through a wide array of alternatives, by also varying diagnosis and treatment levels. The 90-90-90F scenario supposes that it may be easier to reach the 90-90-90 targets for women, while ART scale-up among men may be slower and more challenging, given that diagnosis and ART coverage in most countries is already higher for women than men.

### Cost

In the three scenarios modeled, for all four countries, initial five-year annual program costs are higher with the combined ART and VMMC approach versus ART-only, and then after 2020 are lower with the combined approach versus ART-only. In most of the VMMC priority countries, the scale-up of VMMC is being supported largely by donors.

Donor support is thereby offsetting higher near-term costs and in turn enabling countries to transition to lower sustained ART and VMMC program costs in the future. (See Investment Profile tables in the FY 2015 PEPFAR Country Operational Plan Strategic Direction Summaries for VMMC priority countries, available at http://www.pepfar.gov/countries/cop/c69471.htm. In most cases, government contributions of physical infrastructure, staff time, and information systems are not included in this reporting, because they are not tracked by the governments through VMMC-specific funding streams).

### Limitations

Many limitations of this analysis apply in general to all modeling exercises. The models are parameterized by fitting to data; it is possible that different combinations of parameters would fit the data equally well. The data populating the models are subject to uncertainty. Baseline male circumcision rates, based on survey data, are known to be overreported [18]. Current (2015) ART coverage levels are determined from national program databases, which may contain inconsistencies in data quality. Feasibility of future ART and VMMC scale-up is unknown.

The unit costs for ART and VMMC were not derived using the same methodology, so they may not be exactly comparable. In addition, the unit costs were not varied over time in the model. The cost-related limitations are the ones most likely to affect the conclusions of the paper. If the actual cost of VMMC is significantly higher compared to the annual cost of ART, scaling up VMMC may not lead to lower program costs after the 90% target is reached.

While the authors considered impact outcomes under conditions of lower-than-targeted viral suppression, sensitivity analyses of varying circumcision coverage levels were not included, although 90% circumcision may not be reached by 2020. This was primarily because such modeling projections have been previously completed, revealing an inverse linear relationship between circumcision coverage and HIV incidence [21]. To the extent circumcision targets may not be reached, the additive benefits to epidemic impact from treatment under all scenarios would be less than in the current analyses, as well.

Additional limitations include the following: the model does not address provision of VMMC to subpopulations of males based on age, risk group, behavior, or geography. All males ages 15–49 are included equally in the MC coverage targets. Subnational variations in scale-up of VMMC and ART were not considered in this analysis. Further studies would be necessary to understand the impact of these additional factors on the conclusions.

### Implications

More than 30 years after the identification of the virus that causes AIDS, HIV prevention technologies—if sufficiently scaled in combination—are available to effectively end the pandemic within a generation. However, as articulated by UNAIDS in its Fast-Track Strategy documents, realizing the goal of reducing new infections to 200,000 annually by 2030 requires achievement of a combination of strategies in concert, including 73% viral suppression in both men and women by 2020, increased to 86% by 2030, and approximately 90% MC coverage by 2020, sustained at 90% through 2030. While the combined targets are theoretically possible, current global HIV funding is insufficient to scale up all at once, meaning difficult programming decisions are on the horizon.

Mathematical models are signposts of the way forward, using calculations to forecast the aforementioned five- and 15-year coverage thresholds to achieving an AIDS-free generation. Epidemics occur outside of the lab, however, affecting millions of individuals who do not behave the same way or as predicted or advised. People the world over experience delays in HIV diagnosis and treatment, as well as imperfect care, retention, and ART adherence [22–26], known to result in periods of viremia and increased HIV transmission risk [7,27,28]. Men especially exhibit poorer health-seeking behaviors, including HIV testing [29], which has important implications for Fast-Track strategies, because success is proximally dependent upon 90% HIV diagnosis. Challenges persist with HIV testing and early diagnosis, particularly in men; meanwhile, more than 10 million males have been circumcised to date.

Though models describe the collective potential to end HIV and AIDS, realizing this goal will require a sea change in health-seeking behaviors and the way HIV services are delivered. As donors and partner governments recalibrate HIV programs toward Fast-Track strategies, resource allocations should be sensitive to variations in infection risk, people’s choices, budget realities, program feasibility, and cost-effectiveness projections. Treatment affords reduced morbidity, mortality, and viral transmissibility to both women and men, but must be taken early and consistently and clinically monitored for a lifetime to effect maximum benefit. Male circumcision conveys almost immediate substantial risk reduction to men for life after a single treatment.

## Conclusions

A variety of primary and secondary prevention interventions are available now that place an AIDS-free generation within reach. Modeled scenarios in this paper show that the highly durable and effective male circumcision intervention reduces HIV incidence and long-term costs compared with treatment-only strategies, including if universal levels of viral suppression in men and women are not achieved by 2020. The muted impact of treatment on HIV incidence, resulting from suboptimal viral suppression for any number of reasons, may be restored by achieving high male circumcision coverage. As has been noted by others, in high-incidence generalized epidemics, VMMC is also the most cost-effective option for preventing HIV in the first place [30]. In the context of 90-90-90, prioritizing continued successful scale-up of VMMC increases the possibility of future generations not only free of AIDS but also HIV.

## Supporting Information

S1 Appendix Model-fitting parameters. (XLSX)

S2 Appendix VMMC coverage model inputs. (XLSX)

S3 Appendix ART coverage model inputs. (XLSX)

S1 Fig Model fits of HIV prevalence compared with HIV prevalence data from national Spectrum/AIM files for each country [12]. (a) Lesotho; (b) Malawi; (c) South Africa; (d) Uganda. Blue triangles represent the HIV prevalence input data to which the model was fit; red lines represent the modeled HIV prevalence curves.
(TIF)

S1 Table Annual number of people on ART. (DOCX)

## References

[1] UNAIDS. Fast Track: Ending the AIDS epidemic by 2030. Geneva, Switzerland: United Nations Programme on HIV/AIDS; 2014.

[2] De Cock KM, Jaffe HW, Curran JW. The evolving epidemiology of HIV/AIDS. Aids. 2012 6 19;26(10):1205–13. 10.1097/QAD.0b013e328354622a 22706007

[3] UNAIDS. How AIDS changed everything—MDG6: 15 years, 15 lessons of hope from the AIDS response. Geneva, Switzerland: United Nations Programme on HIV/AIDS; 2015 http://www.unaids.org/en/resources/documents/2015/MDG6_15years-15 lessonsfromtheAIDSresponse, accessed November 16, 2015

[4] Weiss HA, Halperin D, Bailey RC, Hayes RJ, Schmid G, Hankins C. Male circumcision for HIV prevention: from evidence to action? AIDS 2008; 22: 567–574. 10.1097/QAD.0b013e3282f3f406 18316997

[5] Gray R, Kigozi G, Kong X, Ssempiija V, Makumbi F, Wattya S, et al The effectiveness of male circumcision for HIV prevention and effects on risk behaviors in a posttrial follow-up study. AIDS 2012;26: 609–615. 10.1097/QAD.0b013e3283504a3f 22210632 PMC4296667

[6] Kikuyu V, Szolnok L, Garcia MC, Nkonyana J, Curran K, Ashengo T. Voluntary medical male circumcision programs can address low HIV testing and counseling usage and ART enrollment among young men: Lessons from Lesotho. PLoS One 2014;9: e83614 10.1371/journal.pone.0083614 24801714 PMC4011866

[7] Cohen MS, Chen YQ, McCauley M, Gamble T, Hosseinipour MC, Kumarasamy N, et al Prevention of HIV-1 infection with early antiretroviral therapy. N Engl J Med 2011;365: 493–505. 10.1056/NEJMoa1105243 21767103 PMC3200068

[8] Group TAS, Danel C, Moh R, Gabillard D, Badje A, Le Carrou J, et al A trial of early antiretrovirals and Isoniazid preventive therapy in Africa. N Engl J Med 2015;373: 808–822. 10.1056/NEJMoa1507198 26193126

[9] Group ISS, Lundgren JD, Babiker AG, Gordin F, Emery S, Grund B, et al Initiation of antiretroviral therapy in early asymptomatic HIV infection. N Engl J Med 2015;373: 795–807. 10.1056/NEJMoa1506816 26192873 PMC4569751

[10] WHO. Guideline on when to start antiretroviral therapy and on pre-exposure prophylaxis for HIV. Geneva, Switzerland: World Health Organization; 2015. 26598776

[11] Forsythe S, Stover J, Bollinger L. The past, present and future of HIV, AIDS and resource allocation. BMC Public Health 2009; 9 Suppl 1: S4 10.1186/1471-2458-9-S1-S4 19922688 PMC2779506

[12] Stover J, Andreev K, Slaymaker E, Gopalappa C, Sabin K, Velasquez C, et al Updates to the spectrum model to estimate key HIV indicators for adults and children. AIDS 2014;28 Suppl 4: S427–434. 10.1097/QAD.0000000000000483 25406748 PMC4247263

[13] Kripke K, Njeuhmeli, E, Samuelson, J, Schnure, M, Farley, T, Hankins, C, et al. Assessing progress, impact and next steps in rolling out voluntary medical male circumcision for HIV prevention in fourteen priority countries in Eastern and Southern Africa, unpublished. 10.1371/journal.pone.0158767 PMC4955652 27441648

[14] Macro International. Malawi demographic and health survey 2010. Zomba, Malawi: National Statistical Office, and Calverton, Maryland, USA: ICF Macro; 2011.

[15] Macro International. Lesotho demographic and health survey. Maseru, Lesotho: Ministry of Health and Social Welfare; and Calverton, Maryland: ICF Macro; 2009.

[16] Department of Health. South Africa demographic and health survey. Pretoria, Republic of South Africa: Department of Health, Medical Research Council, OrcMacro; 2007.

[17] Uganda Bureau of Statistics. Uganda demographic and health survey 2011. Kampala, Uganda: Uganda Bureau of Statistics (UBOS) ICF International Inc.; 2012.

[18] Thomas AG, Tran BR, Cranston M, Brown MC, Kumar R, Tlelai M. Voluntary medical male circumcision: a cross-sectional study comparing circumcision self-report and physical examination findings in Lesotho. PLoS One 2011;6: e27561 10.1371/journal.pone.0027561 22140449 PMC3226626

[19] Avenir Health. Avenir Health Unit Cost Database. Glastonbury, CT Avenir Health; 2015.

[20] Futures Institute. Goals manual: A model for estimating the effects of interventions and resource allocation on HIV infections and deaths. Glastonbury, CT USA: Futures Institute; 2011.

[21] Njeuhmeli E, Forsythe S, Reed J, Opuni M, Bollinger L, Heard N, et al Voluntary medical male circumcision: Modeling the impact and cost of expanding male circumcision for HIV prevention in eastern and southern Africa. PLoS Med 2011; 8: e1001132 10.1371/journal.pmed.1001132 22140367 PMC3226464

[22] Vital signs: HIV prevention through care and treatment—United States. MMWR 2011; 60(47);1618–1623. 22129997

[23] Marks G, Gardner LI, Craw J, Crepaz N. Entry and retention in medical care among HIV-diagnosed persons: A meta-analysis. AIDS 2010; 24: 2665–2678. 10.1097/QAD.0b013e32833f4b1b 20841990

[24] Mills EJ, Nachega JB, Buchan I, Orbinski J, Attaran A, Singh S, et al Adherence to antiretroviral therapy in sub-Saharan Africa and North America: A meta-analysis. JAMA 2006;296: 679–690. 16896111 10.1001/jama.296.6.679

[25] Paterson DL, Swindells S, Mohr J, Brester M, Vergis EN, Squier C, et al Adherence to protease inhibitor therapy and outcomes in patients with HIV infection. Ann Intern Med 2006;133: 21–30. 10.7326/0003-4819-133-1-200007040-00004 10877736

[26] Rosen S, Fox MP, Gill CJ. Patient retention in antiretroviral therapy programs in sub-Saharan Africa: A systematic review. PLoS Med 2007;4: e298 17941716 10.1371/journal.pmed.0040298 PMC2020494

[27] Attia S, Egger M, Muller M, Zwahlen M, Low N. Sexual transmission of HIV according to viral load and antiretroviral therapy: systematic review and meta-analysis. AIDS 2009;23: 1397–1404. 10.1097/QAD.0b013e32832b7dca 19381076

[28] Quinn TC, Wawer MJ, Sewankambo N, Serwadda D, Li C, Wabwire-Mangen F, et al Viral load and heterosexual transmission of human immunodeficiency virus type 1. Rakai Project Study Group. N Engl J Med 2000; 342: 921–929. 10738050 10.1056/NEJM200003303421303

[29] Cremin I, Cauchemez S, Garnett GP, Gregson S. Patterns of uptake of HIV testing in sub-Saharan Africa in the pre-treatment era. Trop Med Int Health 2012;17: e26–37. 10.1111/j.1365-3156.2011.02937.x 22943376 PMC3443375

[30] Geldsetzer P, Bloom D, Humair S, Barnighausen T. Benefits and costs of the HIV/AIDS targets for the post-2015 development agenda. Health HIV/AIDS Perspective Paper; Copenhagen: Copenhagen Consensus Center; 2015. Available at: http://www.copenhagenconsensus.com/sites/default/files/health_perspective_-_geldsetzer_-_hiv.pdf

