# Correction: Impact and Cost of Scaling Up Voluntary Medical Male Circumcision for HIV Prevention in the Context of the New 90-90-90 HIV Treatment Targets

**DOI:** 10.1371/journal.pone.0169500

**Published:** 2016-12-30

**Authors:** Katharine Kripke, Jason Reed, Catherine Hankins, Gregory Smiley, Catey Laube, Emmanuel Njeuhmeli

There is an error in the Funding statement. The number for the Cooperative Agreement Project SOAR (Supporting Operational AIDS Research) is incorrect. The correct number is OAA-A-14-00060.
