# Supplementary figures and images for: Impact and Cost of Scaling Up Voluntary Medical Male Circumcision for HIV Prevention in the Context of the New 90-90-90 HIV Treatment Targets
Source: PLoS One. 2016 Oct 26;11(10):e0155734. doi: 10.1371/journal.pone.0155734 (PMC5082670; doi:10.1371/journal.pone.0155734)

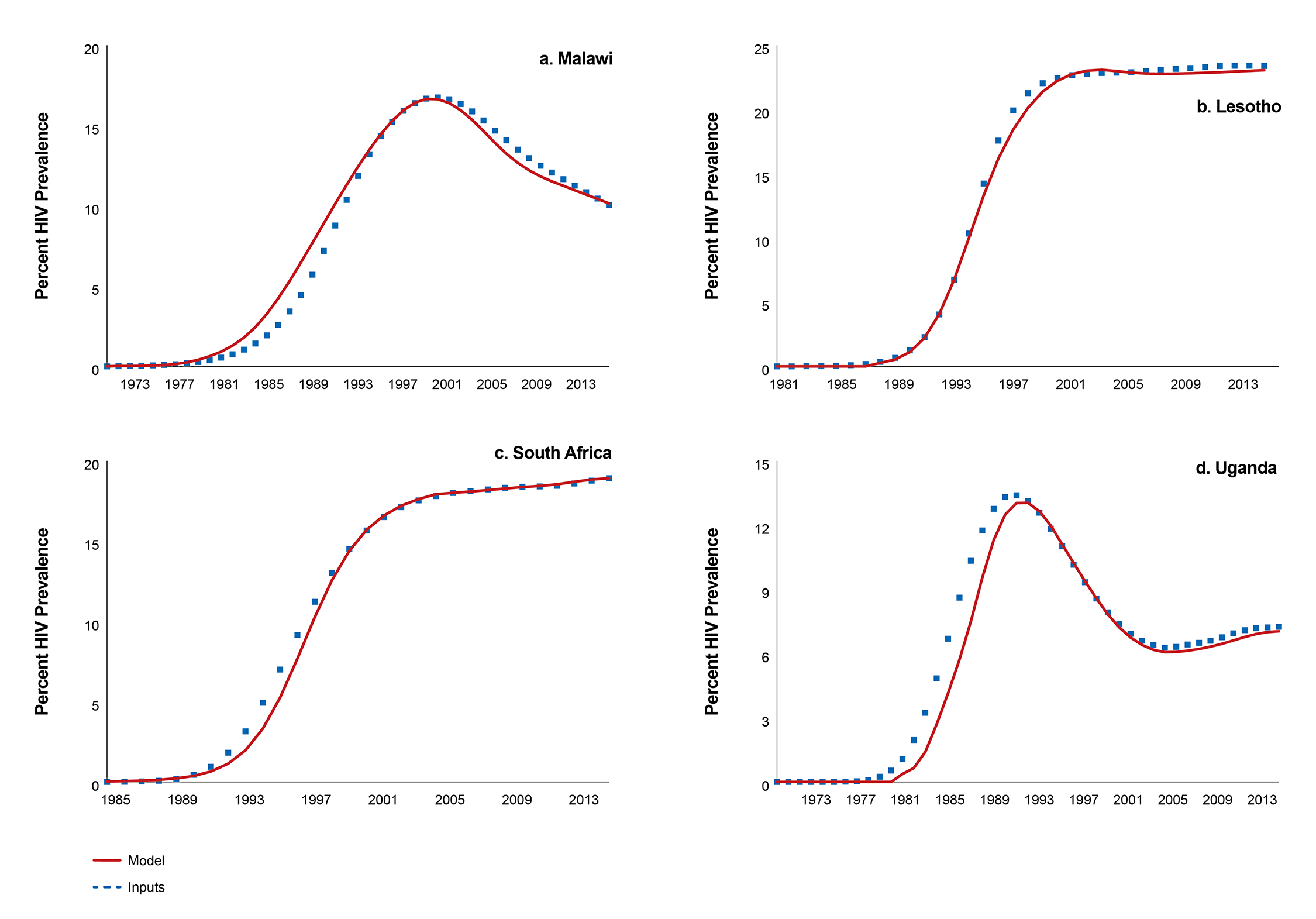

Supplement: S1 Fig — (a) Lesotho; (b) Malawi; (c) South Africa; (d) Uganda. Blue triangles represent the HIV prevalence input data to which the model was fit; red lines represent the modeled HIV prevalence curves. (TIF) [file pone.0155734.s004.tif]
