# Supplementary material for: Impact and Cost of Scaling Up Voluntary Medical Male Circumcision for HIV Prevention in the Context of the New 90-90-90 HIV Treatment Targets
Source: PLoS One. 2016 Oct 26;11(10):e0155734. doi: 10.1371/journal.pone.0155734 (PMC5082670; doi:10.1371/journal.pone.0155734)
Supplement: S1 Table — (DOCX) [file pone.0155734.s005.docx]

Supplemental Table 1. Annual number of people on ART

|  | **Lesotho** | | **Malawi** | | **South Africa** | | **Uganda** | |
| --- | --- | --- | --- | --- | --- | --- | --- | --- |
| **Year** | **90-90-90 no VMMC** | **90-90-90 with VMMC** | **90-90-90 no VMMC** | **90-90-90 with VMMC** | **90-90-90 no VMMC** | **90-90-90 with VMMC** | **90-90-90 no VMMC** | **90-90-90 with VMMC** |
| 2015 | 142,007 | 142,007 | 588,791 | 588,791 | 3,205,407 | 3,205,407 | 813,185 | 813,185 |
| 2016 | 174,815 | 174,815 | 639,446 | 639,446 | 3,461,454 | 3,461,454 | 917,329 | 917,326 |
| 2017 | 213,211 | 212,802 | 717,620 | 716,481 | 3,784,199 | 3,780,124 | 1,033,148 | 1,031,117 |
| 2018 | 248,992 | 247,638 | 774,311 | 770,643 | 4,049,805 | 4,036,921 | 1,152,358 | 1,145,437 |
| 2019 | 288,302 | 285,415 | 836,457 | 828,776 | 4,313,147 | 4,286,631 | 1,280,316 | 1,265,282 |
| 2020 | 327,286 | 322,296 | 896,881 | 883,774 | 4,578,179 | 4,533,774 | 1,409,342 | 1,382,986 |
| 2021 | 334,986 | 328,118 | 917,987 | 899,385 | 4,638,204 | 4,575,581 | 1,462,907 | 1,425,053 |
| 2022 | 340,522 | 331,841 | 934,155 | 910,002 | 4,686,606 | 4,605,687 | 1,509,786 | 1,460,323 |
| 2023 | 345,989 | 335,394 | 949,712 | 919,746 | 4,731,231 | 4,630,991 | 1,556,369 | 1,494,742 |
| 2024 | 351,252 | 338,659 | 964,537 | 928,572 | 4,773,182 | 4,652,805 | 1,602,426 | 1,528,142 |
| 2025 | 356,544 | 341,904 | 979,218 | 937,156 | 4,813,684 | 4,672,665 | 1,648,602 | 1,561,296 |
| 2026 | 361,779 | 345,070 | 993,582 | 945,394 | 4,852,357 | 4,690,490 | 1,694,651 | 1,594,060 |
| 2027 | 366,961 | 348,180 | 1,007,590 | 953,302 | 4,888,841 | 4,706,309 | 1,740,515 | 1,626,440 |
| 2028 | 372,004 | 351,159 | 1,021,057 | 960,755 | 4,922,561 | 4,719,762 | 1,785,706 | 1,658,071 |
| 2029 | 376,875 | 353,980 | 1,033,819 | 967,655 | 4,953,004 | 4,730,487 | 1,829,752 | 1,688,533 |
| 2030 | 381,506 | 356,583 | 1,045,775 | 973,949 | 4,979,579 | 4,738,050 | 1,872,487 | 1,717,682 |
| 2031 | 382,130 | 355,470 | 1,046,742 | 970,237 | 4,952,496 | 4,695,311 | 1,895,000 | 1,728,305 |
| 2032 | 382,525 | 354,059 | 1,046,429 | 965,336 | 4,921,083 | 4,647,996 | 1,916,298 | 1,737,414 |
| 2033 | 383,013 | 352,617 | 1,045,477 | 959,758 | 4,888,570 | 4,598,565 | 1,937,682 | 1,746,018 |
| 2034 | 383,572 | 351,132 | 1,043,834 | 953,466 | 4,854,861 | 4,547,122 | 1,959,151 | 1,754,096 |
| 2035 | 384,176 | 349,591 | 1,041,583 | 946,595 | 4,819,692 | 4,493,709 | 1,980,629 | 1,761,582 |
| 2036 | 384,780 | 347,970 | 1,038,633 | 939,100 | 4,782,791 | 4,438,302 | 2,002,018 | 1,768,385 |
| 2037 | 385,363 | 346,258 | 1,034,989 | 931,022 | 4,743,963 | 4,380,903 | 2,023,227 | 1,774,422 |
| 2038 | 385,901 | 344,444 | 1,030,658 | 922,388 | 4,703,063 | 4,321,514 | 2,044,160 | 1,779,620 |
| 2039 | 386,385 | 342,526 | 1,025,576 | 913,166 | 4,660,000 | 4,260,160 | 2,064,762 | 1,783,943 |
| 2040 | 386,806 | 340,502 | 1,019,739 | 903,370 | 4,614,719 | 4,196,872 | 2,084,983 | 1,787,366 |
| 2041 | 387,159 | 338,376 | 1,013,237 | 893,099 | 4,567,193 | 4,131,694 | 2,104,792 | 1,789,881 |
| 2042 | 387,443 | 336,147 | 1,006,059 | 882,349 | 4,517,424 | 4,064,688 | 2,124,152 | 1,791,483 |
| 2043 | 387,652 | 333,817 | 998,150 | 871,076 | 4,465,414 | 3,995,934 | 2,143,046 | 1,792,172 |
| 2044 | 387,779 | 331,382 | 989,611 | 859,373 | 4,411,216 | 3,925,522 | 2,161,442 | 1,791,934 |
| 2045 | 387,816 | 328,842 | 980,497 | 847,299 | 4,354,903 | 3,853,555 | 2,179,300 | 1,790,755 |
| 2046 | 387,751 | 326,195 | 970,712 | 834,773 | 4,296,531 | 3,780,134 | 2,196,595 | 1,788,635 |
| 2047 | 387,569 | 323,432 | 960,347 | 821,892 | 4,236,175 | 3,705,377 | 2,213,268 | 1,785,545 |
| 2048 | 387,257 | 320,550 | 949,445 | 808,709 | 4,173,942 | 3,629,443 | 2,229,319 | 1,781,513 |
| 2049 | 386,797 | 317,543 | 938,040 | 795,280 | 4,109,981 | 3,552,576 | 2,244,745 | 1,776,568 |
| 2050 | 386,130 | 314,363 | 926,048 | 781,563 | 4,043,836 | 3,474,488 | 2,259,208 | 1,770,466 |
